# The Effect of Various Environmental Conditions on the Impact Damage Behaviour of Natural-Fibre-Reinforced Composites (NFRCs)—A Critical Review

**DOI:** 10.3390/polym15051229

**Published:** 2023-02-28

**Authors:** Muneer Ahmed. Musthaq, Hom Nath Dhakal, Zhongyi Zhang, Antigoni Barouni, Rizal Zahari

**Affiliations:** 1 Advanced Polymers and Composites (APC) Research Group, School of Mechanical and Design Engineering, University of Portsmouth, Anglesea Road, Anglesea Building, Portsmouth PO1 3DJ, UK; 2 Department of Systems Engineering, Military Technological College, Al Matar Street, Muscat P.O. Box 111, Oman; 3 Department of Aeronautical Engineering Technology, Faculty of Engineering Technology and Science, Higher Colleges of Technology, Al-Ain Women Campus, Abu Dhabi P.O. Box 25026, United Arab Emirates

**Keywords:** natural-fibre-reinforced composites (NFRCs), impact performance, environmental conditions, moisture content, damage mechanisms, hybrids

## Abstract

Studies into environmental conditions and their effects on the properties of renewable materials are gaining significant attention in the research field, particularly for natural fibres and their resultant composites. However, natural fibres are prone to water absorption because of the hydrophilic nature of the fibres, which affects the overall mechanical properties of natural-fibre-reinforced composites (NFRCs). In addition, NFRCs are based mainly on thermoplastic and thermosetting matrices, which could be used in automobile and aerospace components as lightweight materials. Therefore, such components have to survive the maximum temperature and humid conditions in different parts of the world. Based on the above factors, through an up-to-date review, this paper critically discusses the effects of environmental conditions on the impact performance of NFRCs. In addition, this paper critically assesses the damage mechanisms of NFRCs and their hybrids by focusing more on moisture ingress and relative humidity in the impact damage behaviour of NFRCs.

## 1. Introduction

Natural fibres, such as plant fibres, dominate manufacturing industries because of their eco-friendliness, low weight, and good mechanical properties [1,2,3,4]. Though natural fibres have a few disadvantages, such as water absorption and restricted maximum processing temperature, they still compete with synthetic fibres, particularly glass fibres, for their superior characteristics, such as specific modulus and elongation at break [5,6]. The mechanical properties of NFRCs depend on the plant’s age, soil characteristics, weather conditions, and harvesting [7,8]. Natural environments, such as water, sunlight, soil, and air, give plant fibres distinctive properties. Despite their differences in composition, all have the same constituents [9]. Plant fibres such as hemp, flax, kenaf, and jute comprise 40–70% cellulose [10]. Cellulose is semi-crystalline, which is responsible for its hydrophilic nature, and possesses excellent mechanical properties [11]. In contrast, hemicellulose is an amorphous polysaccharide comprising five or six carbon rings. The cellulose microfibrils form the cellulose or hemicellulose network. In addition, lignin improves thermal stability and decreases water absorption [12].

More recently, plant fibres have been used successfully for semi-structural and non-structural applications [13]. However, it did not fulfil the full use of structural applications [14]. Perhaps the bast fibres are weak in moisture absorption, which causes them to have low degradation temperatures (less than 200 °C), making them very vulnerable to structural use [15]. Many researchers found that adding coupling agents, compatibilisers, or other chemical modifications could decrease the moisture absorption of NFRCs [16,17,18]. Among the chemical treatments, alkaline is commonly used in the NFRCs, which removes the unwanted constituents such as wax, lignin, and oil substance in the fibre surface and improves the fibre toughness by developing the substantial locking ability of the fibre with the matrix [19,20]. In addition, other coupling agents are also used as a medium to provide excellent interfacial bonding with the fibre and the matrix [21].

The matrix is an essential part of the NFRCs, and it protects the fibre’s surface from mechanical abrasion and provides a barrier against adverse environmental conditions [22]. The most frequently used matrices in NFRCS are thermoset and thermoplastic polymer matrices [23,24,25]. The choice of the matrix in the natural fibre is limited by the temperature because, most of the time, the NFRCs remain thermally unstable to a maximum threshold of 200 °C [23]. Due to the temperature restrictions, the thermoplastic matrix softens the temperature below the maximum threshold and can be easily recycled [11]. Unlike thermoplastic matrices, thermoset matrices are easy to use but tough to recycle and contain harmful chemicals [26]. Thermoset matrices in the NFRCs require high curing temperatures based on the resins used. If the temperature exceeds the maximum threshold of 200 °C, the natural fibres have a significant risk of burning, affecting the component’s structural integrity [27].

Currently, the researchers focus more on the impact test, concentrating on the areas of crashworthiness of vehicles, runway debris, and hand tools falling on composites [28]. In addition, composite laminates are subjected to delamination. However, in NFRCs, water absorption on the surface of the fibres can lead to delamination and cause several factors [29]: (a) poor interface between the natural hydrophilic fibres with hydrophobic organic polymeric matrices; (b) the waxy substance of natural fibres has low surface energy, which makes them bond poorly to polymers. In addition, Dhakal et al. [30] noticed that other factors might be challenging to assess the natural-fibre-reinforced composites subjected to impact tests, such as fibre breakage, matrix cracking, and fibre pull-out.

Environmental conditions of NFRCs constitute a significant concern in structural applications. Natural fibre components like cellulose, hemicellulose, and lignin degrade because of the higher temperatures, resulting in a change in the composite’s mechanical properties [7]. Therefore, a detailed review is needed to understand the environmental conditions of natural fibres and their composites subjected to impact behaviour.

## 2. Chemical Structure and Morphologies of Natural Fibres as Reinforcements

The origin of natural fibre is commonly categorised as plants, animals, and minerals. Plant fibres or bast fibres such as hemp, kenaf, flax, and jute contain cellulose as their primary structural component, whereas animal fibres are composed of proteins. Mineral fibres are not recommended for use because of health-related issues. In addition, plant fibres have much higher strength and stiffness than animal fibres [31]. However, in most cases, geographic factors linked to fibre availability play a significant influence in fibre selection [32]. In Europe, for example, flax fibre has received much attention, whereas, in Asia, jute, hemp, kenaf, ramie, and sisal have gained a lot more. Harakeke fibre (New Zealand flax or *Phormium tenax*) is also examined for structural uses in New Zealand because of its good mechanical properties and local availability [22]. Table 1 depicts the various properties of bast fibres, which differ significantly based on morphological structure, chemical composition, harvesting time, growing conditions, the extraction process, surface treatment, and storage procedure. Pickering et al. noticed that, during optimum harvest time, the strength of the natural fibre drastically reduced to 15% for five days, and the manually extracted fibres increase the strength to 20% over the fibres extracted mechanically [33].

### 2.1. Chemical Structure of Natural Plant Fibre

**Figure 1:**
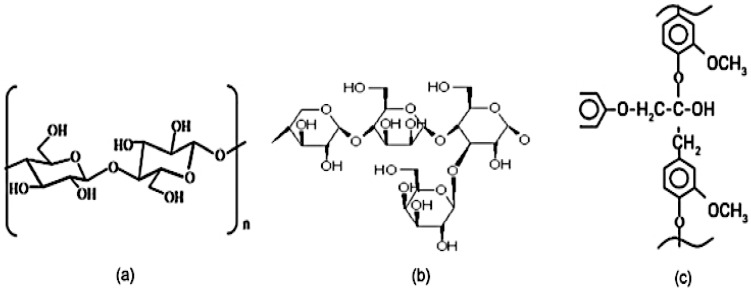
Chemical structures of the main components of natural fibres: (**a**) cellulose, (**b**) hemicellulose, and (**c**) lignin [46].

The chemical structure of plant fibres is composed of cellulose, hemicellulose, lignin, pectin, and wax, as seen in Figure 1. Cellulose consists of D-glucopyranose units connected with β-(1–4)-glucosidic bonds and has a large proportion of hydroxyl groups within the structure [39]. However, the cellulose structure is moisture friendly, which influences the dimensional stability of fibre matrix composites and correlates well with the strength and stiffness of fibre matrix composites [40]. In Figure 1b, hemicellulose polymers are partly soluble in water because they are fully amorphous and likely hydrogen-bonded to cellulose fibrils [41]. There are many surface treatments aimed at eliminating hemicelluloses, but many literature studies have revealed that removing hemicelluloses has a negative impact on various wood properties [42,43,44,45]. In Figure 1C, lignin is amorphous and consists of phenyl propane units, which bind with the hemicellulose within the cell wall. Table 2 illustrates the chemical compositions and moisture content percentage of plant fibres, with kenaf, jute, flax, hemp, ramie, abaca, and sisal the most important for industrial applications.

### 2.2. Morphological Structure of Natural Plant Fibre

**Figure 2:**
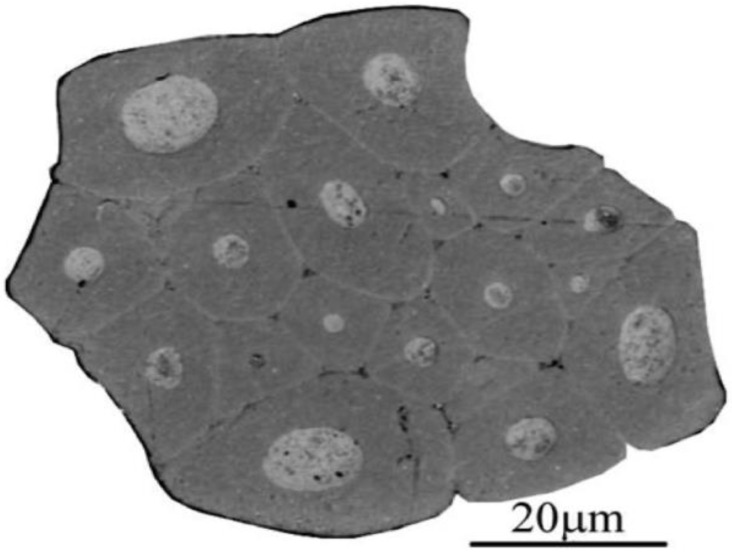
Laser microscope image of the CSA of hemp fibre [50] (reprinted with permission from Taylor & Francis, Licence Number: 501796223).

Natural fibre structure and morphological characteristics are vital because they explain how much improvement in mechanical properties may differ because of variations in the structure, fibre length, volume fractions, microfibrillar angle, and aspect ratio. However, one of the essential underlying assumptions in almost all morphological research and modelling of fibre-reinforced composites is that the fibres have a circular cross-section, and the diameter of the fibres is constant throughout their length [47]. Generally, in natural fibres, the microfibrils typically have a 10–30 μm diameter and are made up of 30–100 cellulose molecules in the shape of an extended chain [48]. Additionally, the mechanical strength of natural plant fibres is primarily influenced by microfibrils; hence, the complex structure of natural plant fibres can significantly affect fibre properties [49]. Figure 2 depicts the complicated cross-sectional shape of natural fibres, which differs considerably from a circle shape. In addition, there are many techniques to estimate the cross-sectional area of natural fibres, and one of the techniques is using an image or photographic analysis [50]. Using an imaging technique, Xu et al. [51] accurately determined the cross-sectional area of sisal fibres. They noticed that this method was able to estimate the cross-sectional area correctly. However, the tests related to imaging techniques were not relevant to tensile tests. Thomason et al. [47] calculated the cross-sectional area of flax and sisal fibres using a digital photograph. They observed that flax fibres have a lower cross-sectional area (CSA) than sisal fibres. In addition, the author further reported a study (refer to Table 3) that the inter-fibre CSA variability is significantly greater than the intra-fibre CSA variability for all types of natural fibres.

**Figure 3:**
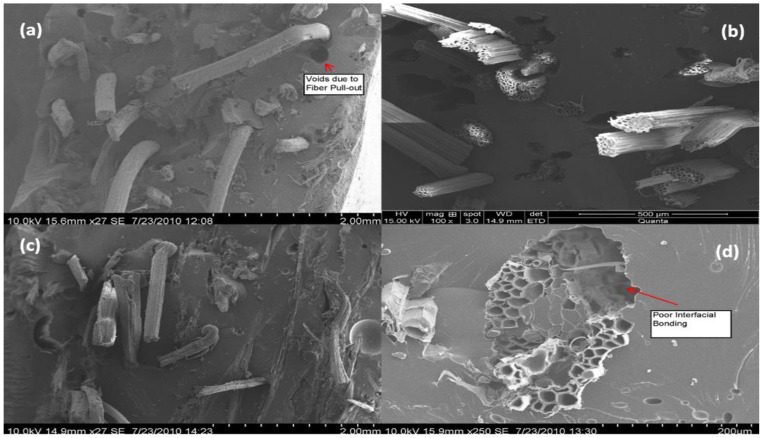
SEM micrographs of fractured surfaces: (**a**) fibre pull-out during the tensile test; (**b**) cluster of bundles during the flexural test; (**c**) fibre breakage during the impact tests; (**d**) poor interfacial bonding [56] (reprinted with permission from Elsevier, License Number: 5446961080043).

Several studies have investigated the morphology of natural-fibre-reinforced composites [52,53,54]. Amongst the studies, scanning electron microscopy (SEM) is the most commonly utilised method for studying the fracture surfaces in plant-fibre composites [55]. Venkateshwaran et al. [56] used an SEM to examine the fractural behaviour and fibre pull-out of banana/sisal-reinforced hybrid composites. They observed that mechanical properties are improved by increasing the fibre length and weight percentage. At the same time, adding fibre causes poor interfacial bonding between the fibre and the matrix. In addition, the SEM analysis in Figure 3a–d shows the formation of voids caused by fibre pull-out.

**Figure 4:**
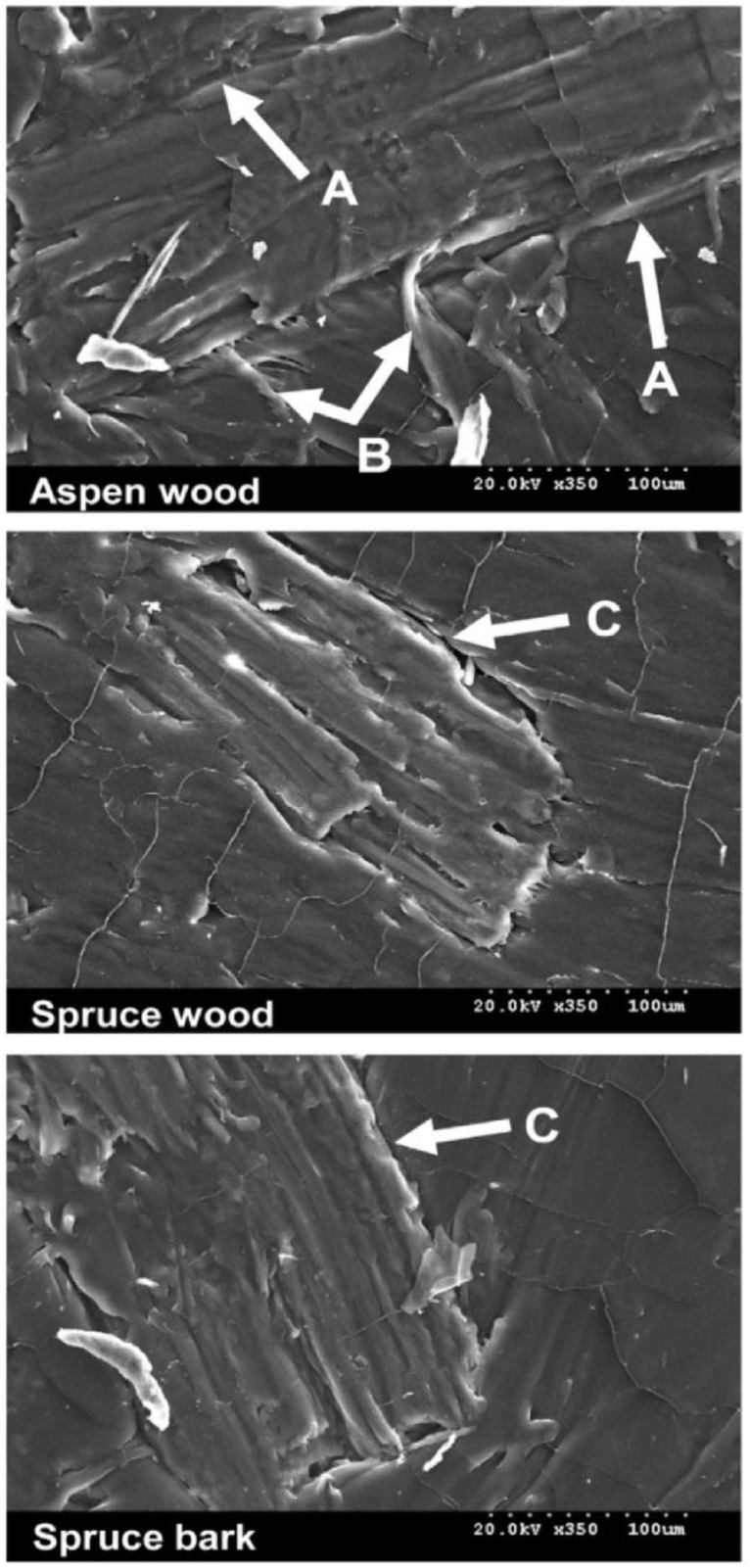
SEM images of aspen wood, spruce bark, and spruce wood fibres: (A) close contact/good wetting, (B) macro-fibrils, and (C) no close contact [57] (reprinted with permission from Elsevier, Licence Number: 5446961305021).

Furthermore, due to the lack of fibre–matrix interaction, the fibres cluster into bundles and become irregularly distributed throughout the matrix, resulting in low mechanical properties. Pothan et al. [52] conducted SEM to investigate the effect of fibre content on the morphology of a random-oriented banana-fibre/polyester composite. They reported that the composite with 40% fibre content had good fibre/matrix bonding, whereas debonding of fibre/matrix was observed in composites with 10% and 20% fibre content. Migneault et al. [57] studied the SEM behaviour of various plant fibres. They noticed the differences in wetting at the fibre–matrix interface of composites between the different fibres used and found that aspen fibres are thoroughly wetted (refer to Figure 4A), whereas, in Figure 4C, spruce and bark fibres are not in contact with matrix HDPE. In addition, SEM micrographs revealed variations in interfacial adhesion and mechanical interlocking, in which aspen fibres had macro-fibrils on the surface interlocked with the polymer matrix, resulting in increased fibre reinforcement (refer to Figure 4B). However, the author concluded that aspen-wood fibres have superior stress transfer and outperformed other fibre-reinforced composites.

**Figure 5:**
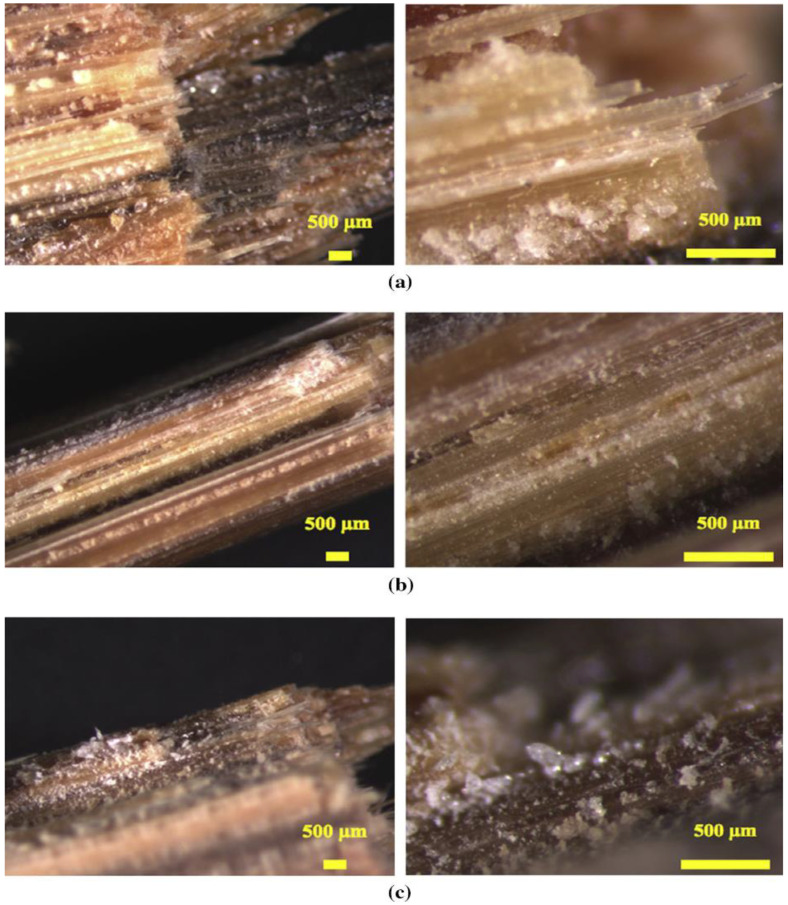
Optical micrographs of the fracture surface unidirectional bamboo-fibre/epoxy composite produced at 100 °C with the pressure of (**a**) 15 MPa, (**b**) 20 MPa, and (**c**) 25 MPa [54] (reprinted with permission from Elsevier, Licence Number: 5446970018062).

On the other hand, Hebel et al. [54] used optical microscopy of the fractured surface to examine the morphology of a unidirectional bamboo-fibre/epoxy composite. As shown in Figure 5, the composites are moulded with autoclave compression at 100 °C with pressures of 15, 20, and 25 MPa, and they observed that the composite generated by 20 MPa pressure has better tensile strength than those produced by 15 and 25 MPa. In addition, they found that lowering the pressure to 15 MPa caused the development of big resin beads on the fibre surface, which resulted in a less wetting and homogeneous covering of the fibres. George et al. [58] investigated the impact of the fibre treatment on the nanostructure of the hemp fibre by using an atomic force microscope (ATM). They highlighted that the surface topography of the hemp fibre was cleaned and the fibre bundles were visible. Similar observations were obtained by Lee et al. [59] for the kraft-fibre-reinforced composites.

## 3. Effects of Various Environmental Conditions on the Impact Toughness Behaviour of NFRCs

Natural-fibre-reinforced composites are increasingly used in various engineering applications such as in aerospace, automotive, and manufacturing industries. When these composites are exposed to harsh environments for an extended period, their performance may suffer. Although temperature and moisture are known to affect fire/matrix stress distributions and matrix properties, the exact nature of the effect of such conditions on the durability of any specific material, mainly woven materials, is generally unknown. However, the following sections will discuss various aspects of the environmental conditions on the impact toughness behaviour of NFRCs.

### 3.1. Factors Affecting the Impact Damage Behaviour

#### 3.1.1. Influence of Moisture Absorption

Moisture absorption causes the fibre–matrix interface region to degrade, resulting in poor stress transfer efficiency and a loss in mechanical and dimensional properties [60]. However, moisture absorption in natural fibre affects the laminates in various ways, such as wear rate, crack propagation, and sliding surface reaction [61]. In addition, water intake affects natural fibres in two ways: (i) the fibre itself swells, and (ii) the density of the fibre changes according to the weight of absorbed moisture [62]. The polar and hydroxyl groups contained in natural fibres are responsible for the water uptake of natural composites. Besides, swelling developing from moisture absorption causes delamination to the laminates, debonding between fibre and matrix, and substantial damage to the polymeric matrix [63,64,65,66,67]. However, the slight swelling of fibres has a favourable influence on natural fibre composites’ mechanical and impact properties because it highlights the mechanical interlocking between the matrix and fibre [68]. In addition, water uptake increases the mobility of side groups and molecular chains, resulting in reversible plasticisation of the polymer matrix [69,70,71,72]. As a result, matrix plasticisation enhances fracture resilience while diminishing the strength, stiffness, and durability of natural fibre composites [73,74,75,76,77,78,79,80,81,82]. According to Chow et al. [83], the modulus and strength of the sisal fibre polypropylene-reinforced composites decreased with increased immersion time in the water. At the same time, the impact strength also increases with the immersion time. Thus, the swelling action of the reinforcing fibre and a plasticisation at the fibre–matrix interface can explain the difference in behaviour between impact and tensile strengths.

In moisture absorption, the most prevalent faults are pores, micro-cracks, and delamination, which could develop during the ageing process. The properties of the composite material will be significantly reduced after ageing [84]. According to Duigou et al. [85], Young’s modulus and the tensile strength of flax/PLA biocomposites decreased with ageing, indicating a linear relationship between water uptake and mechanical property loss. Athijayamani et al. [86] investigated the influence of water absorption on sisal-fibre-reinforced hybrid polyester composites and found a drastic reduction in flexural and tensile strengths during ageing conditions. In addition, Zain et al. [87] investigated the impact and mechanical properties of pseudo-stem fibre-reinforced composites. They highlighted an improvement in flexural and impact strength after ageing treatment, but in tensile test results, there is a reverse effect on the strength of the composites. Concerning the impact tests, the author reported that ageing specimens had enormous average break energy and a higher standard deviation than non-ageing specimens in impact tests.

**Figure 6:**
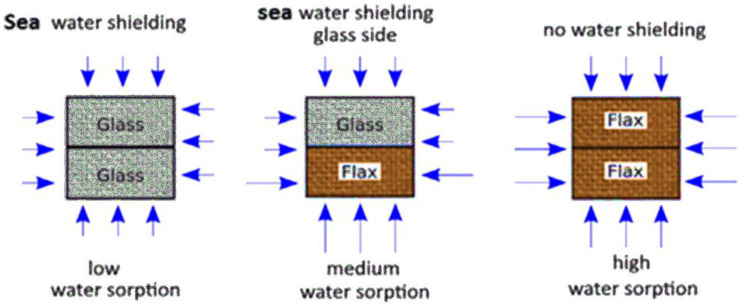
Water diffusion techniques on fibre-reinforced composite materials [88] (reprinted with permission from Elsevier, Licence Number: 5446951281414).

The NFRCs can be vulnerable to chemical attacks from the surrounding environment, weathering, and other natural and ageing degradation [88]. Besides, the chemical from seawater corrodes the fibre’s surface and causes a small gap between the fibre and the matrix. Figure 6 depicts the various ways seawater can enter synthetic/synthetic, natural/natural, and synthetic/natural FRP hybrid composites. In numerous applications, fibre-reinforced composites are often subjected to environmental and chemical attacks. The composites based on natural fibre usually degrade more under environmental and chemical conditions. For instance, Narendra et al. [89] examined the impact and compressive strength of hybrid coir pith/nylon/epoxy composites under seawater ageing. They found that chemical-treated coir pith/nylon-reinforced composites achieved incredible impact and compressive strength when the samples were immersed in seawater for 31 days. In addition, they stated that the moisture uptake of treated composites was lower than the untreated composites because of the composites filled with seawater forming voids, cracks, and microvoids in the surface of the composites. In another study, Le Duigou et al. [90] observed that the moisture behaviour of seawater in flax/poly(lactic acid) composites had several degradation mechanisms, such as debonding or fibre pull-out from the fibre/matrix interface, swelling of the fibres, and reduction in mechanical properties. In addition, he added that water is hardly affected by the rigidity of unreinforced poly(lactic acid). However, biocomposites gradually lose their tensile strength and stiffness with the water entering the material.

#### 3.1.2. Effects of Humid Conditions on the Performance of Natural-Fibre-Reinforced Composites

Natural-fibre-reinforced composites offer many advantages in engineering applications, particularly in the manufacturing industries where they consider the composites as low weight, less in cost, and easily renewable. To spread the use of natural fibre composites in many industries, several issues must be addressed, including the insufficient adhesion between the hydrophobic matrix and fibres, low wettability of non-polar polymers, and significant water uptake [2,91]. The structure of plant fibre is composed of cellulose, hemicellulose, lignin, pectin, and wax, in which lignin provides efficient protection from adverse environmental conditions such as humidity and temperature [92,93]. Besides, NFRCs are hydrophilic and are commonly subjected to different weather conditions during their lifetimes. However, under humid conditions, plant fibre achieves a high level of moisture absorption with a higher voids content, resulting in structural modification of the fibres and a change in the mechanical and impact properties [94].

**Figure 7:**
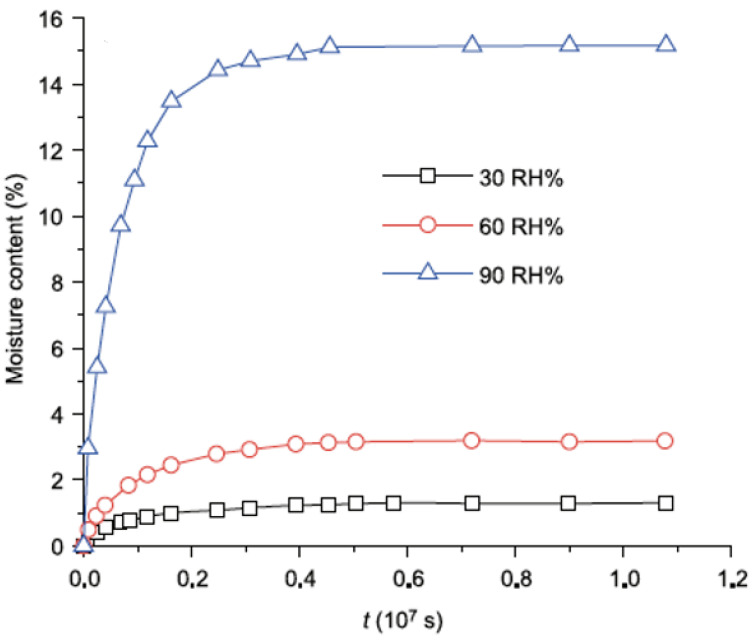
Experimental results on moisture absorption of sisal-fibre-reinforced composites with different relative humidities [96] (reprinted with permission from John Wiley and Sons, Licence Number: 5495871226329).

Extensive efforts have been made to investigate the modulus and swelling deformation of NFRCs by using water-bath experiments, which will speed up the process of moisture absorption and mechanical degradation [95,96,97,98]. The relationship between the modulus and humidity absorption can be determined in these experiments. However, the investigation does not simulate the natural environment with varying relative humidity [99]. In addition, the mechanical performance of NFRCs would affect not only relative humidity but also temperature changes. Alvarez et al. [96] studied the mechanical and moisture absorption of sisal-fibre-reinforced composites in different relative humidity environments. They observed that moisture content monotonically increases with time until it reaches equilibrium. As shown in Figure 7, the relative humidity (RH) is adjusted to 30%, 60%, and 90%, and a high relative humidity speeds up moisture absorption and raises the equilibrium moisture content. David and Bruce [100] experimented with the mechanical characteristics of flax-fibre-reinforced composites by considering relative humidity as a concern. They noticed that Young’s modulus decreased when the relative humidity varied from 30–80%. This approach is also underlined by Symington et al. [101] for flax fibres. The drop in Young’s modulus above a specific moisture content threshold was affected by fibre plasticisation. However, in other studies, the researchers noticed that Young’s modulus of natural fibre increases with the relative humidity up to a specific threshold of water absorbed [39,101]. For example, Placet et al. [39] found that Young’s modulus in hemp fibres increased up to 20% with a 25–80% relative humidity range. In addition, the author noticed that the increase in elastic modulus could be due to the reordering of microfibrils and the adjacent molecules acting as a matrix. This reordering could be caused by swelling of the fibres. Furthermore, the formation of chemical bonds in the cellulose and lignin complex molecular network may increase the material’s flexibility and compliance.

The literature’s findings on the effect of water uptake on tensile strength are consistent. However, RH often causes a rise in stress during failure, up to a maximum value of 50 to 60% of RH [39] or 70% of RH [102], which results in a drop in tensile strength. This is because water absorption within the fibre can cause a fracture of the hydroxyl groups between the amorphous region matrix and the crystalline portion of the fibre. In a similar study, Scida et al. [103] discovered that hygrothermal ageing influences the tensile characteristics of flax composites. Young’s modulus was reduced by 33% in the first three days at 90% RH and 55% after 38 days. In addition, the author further investigated the interfacial strength of a single flax-fibre/epoxy micro-composite and observed that when the composites were immersed in water for 135 h, their shear strength was reduced by 60%. This results in the reduction in interfacial strength caused by the swelling of fibres at the fibre–matrix interface. Moudood et al. [104] investigated the effect of moisture uptake in flax-fibre-reinforced composites. They found that composite panels with flax fibres from 70 to 95% relative humidity (RH) showed significant warpage because of high moisture content. In addition, the fibre–matrix contact became weaker, and porosity in the microstructure of the composites increased. Though the fibre–matrix interface was altered, composites manufactured with 50% RH-conditioned fabrics had the best tensile strength, whereas composites below and above that value had lower tensile strength. According to the researchers, the water molecules in the fibres are plasticised and distorted, increasing the strain at break and lowering young’s modulus.

Unlike the studies mentioned above, which focussed on the effects of varied environmental conditions on the flexural, tensile, and other mechanical properties of NFRCs, further research is still needed on the impact behaviour and shear responses of natural composites’ varying RH values.

#### 3.1.3. Influence of Matrix Properties on the Moisture Ingress Behaviour of NFRCs

Moisture ingress is present in all organic matrices. Generally, organic matrices are permeable to a broad spectrum of organic liquids, resulting in a decrease in matrix modulus [105]. In addition, they are unable to resist extreme temperatures. However, certain resins are more resistant to dilute acids and alkalis, which is better than stainless steel or alloys. This is the main reason composite materials outperform solids in corrosive resistance [106]. Hydrolysis is the most common type of chemical degradation in matrix materials, in which water, OH, H+, or H30+ ions attack chemical groups inside the matrix [107]. In acidic or alkaline settings, the hydrolysis reaction is more severe. The polar groups within polymers, particularly the ester, amide, carbonate, and amide, are the most susceptible to hydrolysis [108]. Besides, chemical oxidation with oxidising acids such as nitric, sulfuric, or other oxidising agents like peroxides and hypochlorite is another primary form of chemical deterioration in the matrix material. Active free radicals, such as H_2_O and HO, target the polymer’s primary chain bonds. Therefore, polyesters have more ester groups than other resins and are, thus, more sensitive to hydrolysis, particularly in alkaline settings [109].

In wet conditions, water absorption of the matrix material compromises the mechanical stability of advanced composites [110]. Notably, polymer resins from hydrophilic groups absorb water molecules in natural fibre, causing a change in the matrix’s physical and mechanical properties. Plasticisation, for example, occurs at various stages when there is an interaction of absorbed water molecules with the matrix, resulting in a degradation of fibre and matrix interface bonding, microcracks, chain scission, and a decrease in mechanical properties [111]. In addition, plasticisation decreases the glass transition temperature (T_g_) [111]. However, to enhance the glass transition temperature (T_g_), the water absorption rate in polymer matrices must increase with the increasing temperature [112]. Therefore, increasing the temperature improves the segmental mobility and achieves a higher activation zone, enhancing water absorption in the polymer matrix [113]. Apicella et al. [114] investigated the moisture behaviour and mechanical properties of polyester resins such as vinyl ester, bisphenol, and isophthalic. They noticed that vinyl ester had the highest equilibrium water uptake of 65% at 20 °C and isophthalic resin had the lowest at 0.35%. This shows that isophthalic resins with the highest ester content had the least hydrolytic stability. Concerning tensile tests, the authors further observed that isophthalic resin lost 32% of its tensile strength, 11% of its tensile modulus, and 18% of its elongation to break after 50 days in water at 20 °C. In another study, Agarwal and Broutman [115] proved that moisture content has no significant effect on fibre-dominated properties but may reduce matrix-dominated properties. Browning et al. [116] also observed a similar study in unidirectional carbon epoxy laminates. They noticed that the strength of the matrix decreases by increasing the moisture content. In addition, the moisture lowered the elastic modulus and strength of the composites in transverse tensile and shear loading, whereas the axial properties were unaffected. Harper [117] found that polyester matrices have excellent resistance to acids and distilled water for extended periods at temperatures as high as 210 °F. In addition, they also observed the water behaviour of glass mat laminates treated with polyester matrices and found that during dry conditions, the ultimate strength was up to 89.7 MPa, which was significantly higher than the wet conditions of 81.4 MPa.

Another essential feature in the moisture absorption of the polymer matrix is plasticisation, which occurs because of the absorption of water molecules. Small molecules in small solutes disrupt the intermolecular connection between polymer chains, making chain movement easier. As a result, the polymer’s glass transition temperature (T_g_) decreases. T_g_ reduction can have a significant effect on the composite’s characteristics. However, this mechanism occurs mainly in the amorphous region, which is more prominent for glassy polymers [118]. Pipes et al. [119] emphasised that the greater the degree of plasticisation, the higher the equilibrium solubility. Besides, plasticisation could result in a significant loss of stiffness and increased creep rate and diffusion coefficient.

Moreover, no single theory or model has enough experimental evidence to describe all hygrothermal occurrences. Besides, several articles reported on the hydrolysis behaviour of polymer matrices, particularly thermosets in synthetic fibres, and no studies were reported on NFRCs and thermoplastic polymer matrices. Though the moisture ingress mechanism on polymer matrix is identical for all the fibre composites, polymer matrix embedded with natural fibres has yet to be focussed on in the research field.

### 3.2. Mechanisms of Moisture Ingress in NFRCs

**Figure 8:**
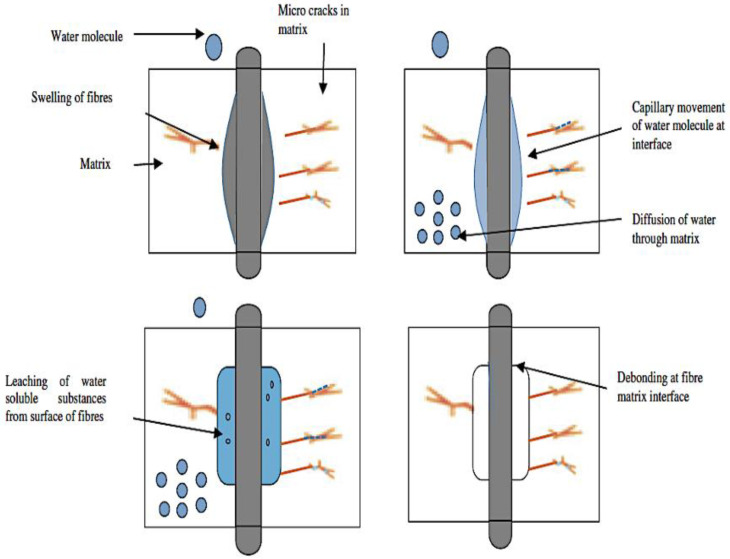
The sequences of NFRCs’ structural integrity loss caused by water absorption [120] (reprinted with permission from SAGE Publications, Licence Number:5446970811502).

One of the most critical challenges in NFRCs is degradation when exposed to environmental conditions such as humidity, high temperature, and water [120]. It is evident that moisture ingress substantially affects the mechanical properties of NFRCs [121]. Two main mechanisms can describe the moisture ingress of natural fibre: (a) linear Fickian behaviour, where the maximum weight of water gradually reaches equilibrium after a swift initial take-off, and (b) pseudo-Fickian behaviour, in which the maximum weight of the water does not reach equilibrium after take-off [62]. Figure 8 depicts the sequences of the NFRCs’ structural integrity loss caused by water absorption. The increased moisture absorption aids microbial attack, resulting in a process known as biodegradation [122]. Besides, the influence of moisture on the mechanical behaviour of the NFRCs must highlight the need to precisely anticipate absorption behaviour and humidity content, mainly for structural load-handling applications designed for extended life [123]. Owing to its flexibility and numerical tractability, the 1D (one-dimensional) Fickian diffusion model is most frequently used to predict absorption and moisture content [124]. Though Fickian behaviour is significantly used during the first moisture uptake, polymeric composites such as natural and synthetic fibres exhibit non-Fickian absorption behaviour in the long run [125,126,127,128,129,130]. The non-Fickian behaviour is also known as pseudo-Fickian because it is observed anomalously after equilibrium, where the excessive level of moisture ingress is significantly reduced [131]. Moreover, NFRCs can exhibit Fickian and non-Fickian behaviours at different temperatures or be exposed to other environmental conditions [132].

#### 3.2.1. Fickian and Non-Fickian Behaviours

Several researchers produced different models to study the moisture ingress behaviour of polymeric composites, in which the overall performance was modelled by considering the diffusion mechanism [133,134,135,136]. Equations (1) and (2) describe Fick’s law, frequently applied to the steady-state one-dimensional diffusion model for simplicity and mathematical traceability [120].

(1) $$J=-D\;\frac{dC}{dx}$$

(2) $$\frac{dC}{dt}=-D\;\frac{dC^{2}}{dx^{2}}$$

where J is the flux laminate, which is the flow of matter per unit area per unit time, *D* is the diffusivity or diffusion coefficient, and $\frac{dC}{dx}$ is the concentration gradient of the diffusing material. According to Dhakal et al. [137], the following statements represent Fick’s first law of diffusion:

i. The flux (J) through a material unit area is proportionate to the concentration gradient (*C*) measured perpendicular to the material.
ii. The molecular diffusion coefficient (*D*) equals the square of diffusive molecule velocity.

Further to the above assumptions, moisture absorption plays a vital role in water dispersion in the composites during low- and high-concentration regions [138]. However, this behaviour could be exposed to different environmental circumstances, such as humidity and high- and low-level temperatures. Therefore, the total moisture uptake can be expressed in Equation (3) [138].

(3) $$\frac{M_{t}\;}{M_{s}}=\;1-\overset{\infty }{\underset{n=0}{\sum }}\frac{8}{{\left[\left(2n+1\right)\pi \right]}^{2}}exp\;\left[\frac{-D\;{\left(2n+1\right)}^{2}\;\pi^{2}\;t}{h^{2}}\right]$$

where $M_{t}$, denotes moisture uptake at *t* (time), $M_{s}$ denotes the diffusion at the saturated time, *D* denotes diffusivity or coefficient of diffusivity, and h indicates the sample’s thickness.

**Figure 9:**
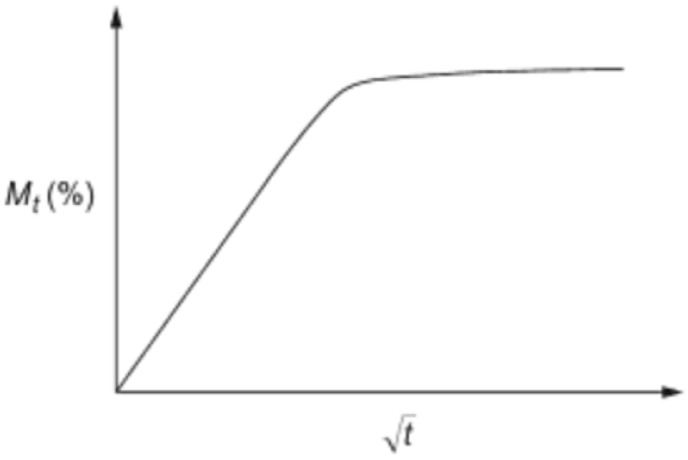
Fickian diffusive curve [137] (reprinted with permission from Elsevier, Licence Number: 5447061247707).

Numerous studies have focussed on the moisture uptake characteristics of polymer composites and determined that Fickian behaviour sufficiently describes the composites’ water ingress properties [139,140,141]. Figure 9 illustrates the Fickian diffusive curve, representing water absorption as a two-step process. During the early phase, sudden ingress occurs as water primarily enters between the phases and progresses consistently [139]. However, in the second phase, the composite material reaches saturation and exhibits a substantial drop and flattening, resulting in swelling and eventually reaching the final equilibrium [140]. To evaluate sorptivity, the relative weight gain, $\frac{M_{t}\;}{M_{s}}$, is displayed as a function of the time’s square root and can be utilised as measurable and comparative assessment results between specimens.

In the beginning, the moisture uptake varies exponentially with the time’s square root, expressed in Equation (4), which determines the Fickian diffusion behaviour.

(4) $$M_{t}=4\;M_{s}\;\sqrt{\frac{D\cdot \;t}{\pi \cdot h^{2}}}$$

The *D* (diffusion coefficient), a fundamental element in Fick’s diffusion model, determines the water molecules’ ability to diffuse into NFRCs. Using Fick’s diffusion model, the average coefficient of diffusivity was calculated using Equations (5) and (6):

(5) $$D=\frac{\pi }{16\;M_{s}{}^{2}}\;{\left[\frac{M_{t}}{\sqrt{t/h}}\right]}^{2}$$

(6) $$D=\;\pi \;{\left(\frac{h}{4\;M_{s}}\right)}^{2}{}^{\;}\;{\left(\frac{M_{2}-M_{1}}{\sqrt{t_{2}}-\sqrt{t_{1}}}\right)}^{2}$$

**Figure 10:**
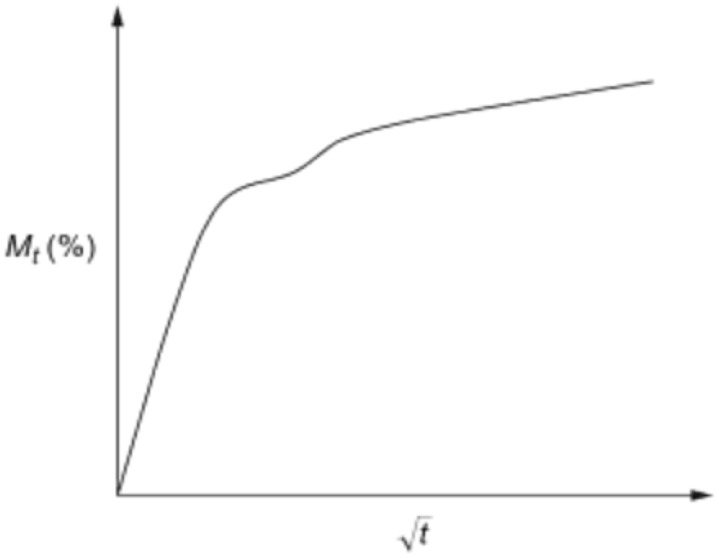
Non-Fickian diffusion behaviour of two-stage absorption curve [137] (reprinted with permission from Elsevier, Licence Number: 5447061247707).

Non-Fickian behaviour is monitored, particularly for higher temperatures, which are commonly characterised based on their appearance in plots of water uptake [142,143,144,145]. The two-stage curves in Figure 10 have two distinct phases [142]. A Fickian response is observed during the early stages of absorption; a slower rate of non-Fickian absorption may have an initial elastic period and then plateau [137]. Therefore, to determine the mass uptake of $M_{t}$ of the entire sample, the curve is derived from the initial portion of absorption, as shown in Equation (7).

(7) $$M_{t}=\;k\;\cdot \;t^{n}$$

Considering that *n* is constant, then the initial absorption stage corresponds to *n* = 0.5, and k is the initial absorption slope of $M_{t}$ versus $\sqrt{t}$.

During the second absorption stage, moisture improves structural relaxation, creating voids, blisters, and retaining water [137]. According to Bao and Yee [146], the two-stage curve can be calculated by the following Equation (8):

(8) $$M_{t}=\;M_{s}\;\left(1+k\;\sqrt{t}\right)\;\left\{1-exp\;\left[-7.3\right]\;{\left(\frac{D\;t}{h_{2}}\right)}^{0.75}\right\}$$

Whereas the other components have already been defined, the $\left(1+k\;\sqrt{t}\right)$ determines the second stage, which is connected to the relaxation rate (k). In the case of *n* = 1, the non-Fickian absorption is considered by 0.5 < *n* < 1. Therefore, Equation (9) can be calculated using the initial slope.

(9) $$k=\frac{4\;M_{s}}{h}\;\;{\left(\frac{D}{\pi }\right)}^{0.5}$$

Moreover, the non-Fickian behaviour mechanism is divided into three distinct categories, as illustrated in Table 4.

#### 3.2.2. Diffusion Coefficient and Influencing Parameters

The most common practice for measuring absorption characterisation is to follow the rules of ASTM standards, which are correlated to the Fickian diffusion model. However, assuming Fickian behaviour, a prior may result in (a) erroneous estimation of extreme moisture absorption, essential in estimating the thermomechanical property losses, (b) ignoring non-Fickian behaviour, which is frequently observed in experiments under long-term absorption, and (c) experiments using thermogravimetric absorption being terminated prematurely [132].

Acknowledging the drawbacks of Fickian theory, many scholars have recommended numerous models based on moisture uptake to study irregular or hindered diffusion behaviour. These encompass the Jacobs–Jones model, also called the dual-diffusivity two-phase polymer model [126,127], a model of hindered diffusion (HDM), sometimes called dual-mode sorption, is based on the Langmuir type [130], and coupled diffusion relaxation models [128]. However, using these models, evaluating the absorption behaviour of specific composite material with material properties under experimental conditions (parameters related to diffusion or absorption) is easy. Glaskova et al. [132] conducted a comparison study to determine how well these models represented the non-Fickian behaviour of an epoxy system. They concluded that Langmuir’s model proved particularly beneficial. A similar observation was made by the other researchers [147,148,149] and found that the Langmuir-framework-based 1D HDM accurately predicted the short- and long-term absorption of moisture in polymeric composites. Besides, the HDM assumes that molecular-sized interstices influence the water absorption in composite laminates. Guloglu et al. [123] explained interstice influences in the water absorption in composite laminates and noticed the following causes: (a) the interstices are reliant on the microstructural shape and cross-link density. Conversely, the affinity between polymer and water is influenced by the hydrophilic functional groups, such as hydroxyl and amine. (b) Some water molecules absorbed are likely to form strong bonds with the polymer’s polar groups and hydrogen bonding sites. In such circumstances, the molecules would no longer be part of the continuing diffusion process, which corresponds to them being bound or immobile. In contrast, unbound or mobile water molecules do not adhere to any physical structure and are free to travel through interstice. (c) In thermogravimetric studies of polymeric materials, rapid early moisture intake is typically followed by a slower absorption rate when bound water molecules hinder diffusion. In addition, it can take a long time for moisture equilibrium to occur, as the exchange rate between bound and unbound water plays a key role. Therefore, it is understood that the discrepancy in these rates reflects several different absorption behaviours on two-time scales and can be successfully modelled by an HDM.

**Figure 11:**
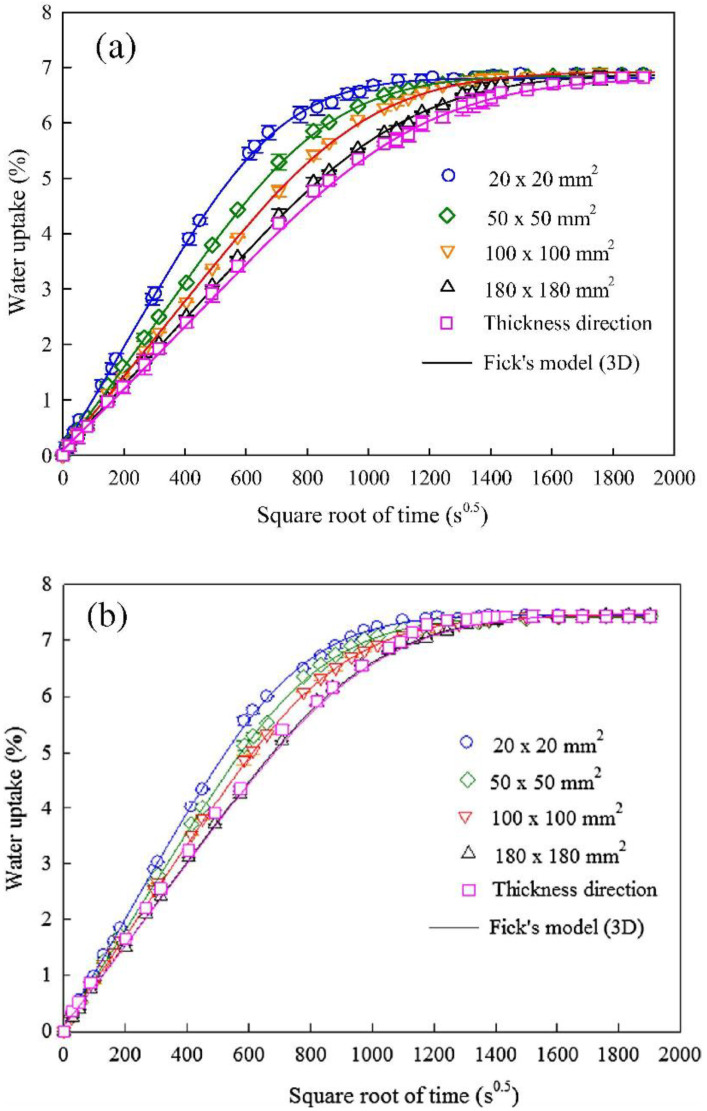
Unsealed samples with equal thickness and four surface dimensions aged in tap water: (**a**) flax–acrylic and (**b**) flax–epoxy [154] (reprinted with permission from Elsevier, Licence Number: 5447070100954).

Several authors used the 3D Fick’s model to identify the diffusion parameters in polymer composites to analyse the kinetics of diffusion [148,150,151,152]. Grace et al. [148] used experimental absorption data to present a new method for characterising polymeric composites’ anisotropic moisture absorption behaviour. Using the 3D Fick’s model, the authors observed that the absorption parameters would provide the best convergence with the experimental data that can be quickly and accurately determined. According to Saidane et al. [153], the morphology and anisotropy of the flax fibre have a substantial influence on the diffusion direction. The 3D Fick’s model predicted diffusion kinetics that agreed well with the experimental curves. A study by Chilali et al. [154] investigated the 3D Fick’s model in flax-fibre-reinforced thermoplastic and thermosetting composites. The absorption curves in Figure 11 found that the equilibrium mass gain grows linearly with fibre orientation, declines with thickness, and is highly correlated to the diffusion rate.

Interestingly, very few experts have attempted to determine absorption behaviour with the exact solution of the HDM or any other Fickian or non-Fickian model [146,147,148,149,150,151,152,155]. This could be due to mathematical difficulties and the intensive processing (computational) work required to restore absorption parameters [156]. According to Guloglu et al. [123], finding the correct solution to find an absorption curve that best fits the experimental data by solving the complicated inverse problem by devising an algorithm search is quite challenging. However, obtaining this set of absorption values may involve many reiterations, depending on the preliminary assumptions and the search algorithm’s convergence rate. Considering this framework, the author also stated that using exact analytical solutions requires a simpler and more accurate computational approach to recover absorption parameters from absorption models.

## 4. Effects of Temperatures on Properties of NFRCs

### 4.1. Influence of Thermal Degradation Caused by Various Temperatures

**Figure 12:**
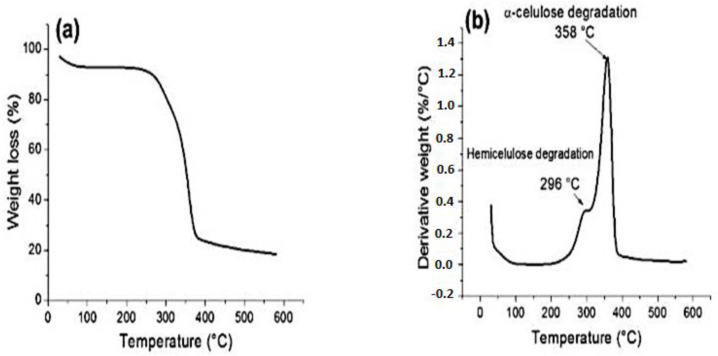
(**a**) TG and (**b**) DTG curves of fique fibres [161] (reprinted with permission from Elsevier, Licence Number: 5494340333464).

The influence of thermal degradation in NFRC plays a vital role in structural applications. The fibre components, such as cellulose, hemicellulose, and lignin, begin to degrade at higher temperatures and, thus, cause a change in mechanical properties [157]. For a better understanding of the thermal degradation of NFC, Table 5 depicts the stages of thermal degradation associated with weight loss. Ray et al. [158] examined the thermal behaviour of jute-fibre-reinforced composites, and they observed two peaks in treated and untreated fibres. The initial peak of untreated fibres at 300 °C signified hemicellulose degradation, and the subsequent peak at 365 °C represented heat degradation of cellulosic content. Besides, the subsequent peaks play a significant role in weight loss because cellulose contributes to most of the natural fibre. However, in contrast to the subsequent peak of untreated fibre, only one peak appeared at a lower temperature. Alabdulkarem et al. [159] investigated the thermal properties of agave fibre and observed a 5% initial mass loss at 221 °C, and a significant weight loss was achieved at 379 °C, with a 64% reduction in mass. The authors further assumed that the thermal characteristics of agave fibres can withstand temperatures of up to 221 °C and can be used in applications where the maximum temperature is less than 221 °C. However, in another comparative study of treated and untreated fibres, the authors Nassir et al. [160] reported that the thermal stability of treated fibres improved from 449 to 491 °C because of its high crystallinity index. In addition, it was found that treated fibres improve thermal stability by increasing fibre crystallinity. In another study, Hidalgo et al. [161] examined the thermal properties of fique-fibre-reinforced polyethylene liner (LLDP) and epoxy composites. They observed the degradation occurred at 296 °C with a mass loss of 20%, as shown in Figure 12a,b. They further noticed at 170 °C that the presence of epoxy resins and polyethylene liner helps to reduce the fibre composites’ thermal stability.

**Figure 13:**
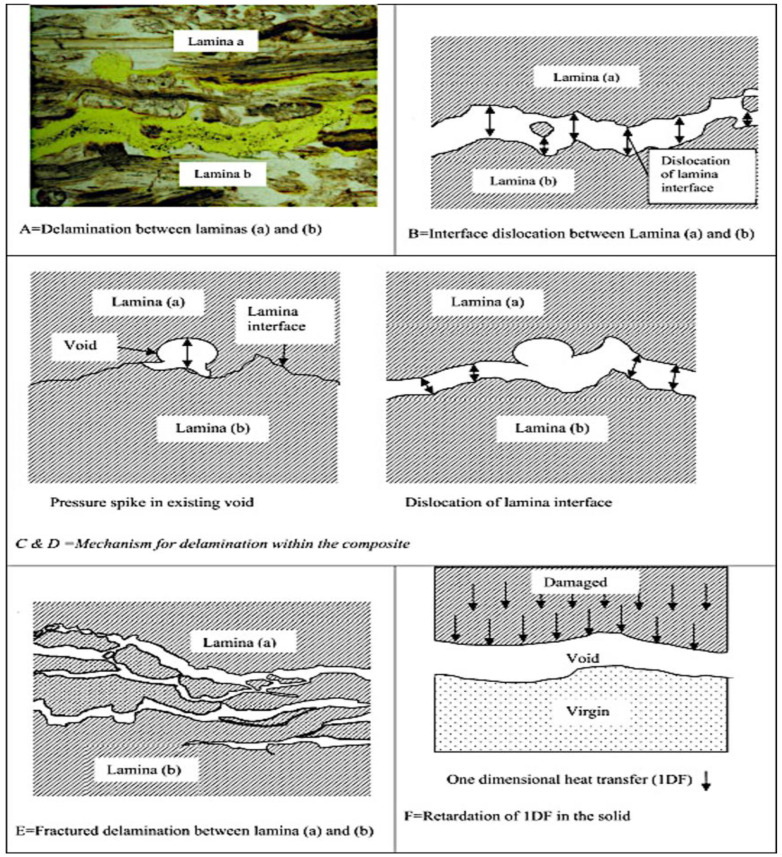
Delamination mechanisms observed in composite due to various environmental conditions [164] (reprinted with permission from Elsevier, Licence Number: 5446951486229).

Concerning impact and mechanical properties, the NFRC was greatly influenced by the post-curing temperature and the exposure temperature. For instance, the damage threshold load in flax/epoxy composites decreased with increasing post-curing temperature [162]. According to Ma et al. [163], heating flax fibre to 180 °C changes its thermochemical composition, resulting in a loss of tensile strength in both the fibre and the matrix. Contrastingly, the mechanical properties of the polymer matrix are strongly dependent on the temperature because the temperature rise makes the transitions from a rigid, glassy state to a soft, rubbery one. In addition, Fan et al. [164] discovered that the small voids in the natural fibre indicate micro-delamination, which appears to be the start of more significant delamination. As shown in Figure 13A, a vast horizontal void perpendicular to the direction of heat flow indicates that delamination between the lamina in a composite may result from decomposition. This could be owing to shear stress created by the various deformations between fibre and matrix or the gas pressure. As illustrated in Figure 13B, each contour of the void region coincides with each opposing contour, implying that the void has opened up during thermal degradation and that previously conjoined contours have become dislocated. This could be due to thermal movement, but it is more probably due to the accumulation of gases, mainly steam, which would cause a pressure rise in that region. In addition, the authors further noticed in Figure 13C,D that the gas pocket temperature starts to dislocate the matrix from the fibres. This dislocation would follow the path of less resistance, thereby forcing a separation between two laminates in the relatively weak interface. In some cases, delamination is shown to prevent the thermal degradation of lower-lying material. The void seems to have an isolating effect by inhibiting thermal transfer in the solid, as shown in Figure 13E,F.

### 4.2. Effects of Temperatures on Impact Damage Behaviour of NFRCs

NFRCs are widely used in automotive, aerospace, and marine applications because of their excellent stiffness and strength-to-weight ratios. However, these materials are particularly vulnerable to low-velocity impact damage [167]. The majority of studies focus on impact energies that are significantly higher than the barely visible impact damage (BVID) energy. Nonetheless, it is critical to note that even extremely low-energy impacts can cause scarcely visible damage, resulting in loss of impacted laminated properties [168,169]. Besides, the damage from low-velocity impact will occur at low or high temperatures in servicing conditions. An instance would be a tool dropping on the aircraft wing or a service vehicle hitting the side of the fuselage in a tropical climate with high temperatures [170]. As a result, it is critical to comprehend the impacts of temperature on impact resistance, which has gotten little attention in the literature. In addition, there are no sufficient studies on the effects of temperature on the impact damage behaviour of NFRCs. Therefore, it is essential to understand the influence of high, low, and cryogenic temperatures that affect the impact characteristics of natural- and synthetic-fibre composites by studying the various authors’ works in the following section.

#### 4.2.1. Effects of Cryogenic Temperatures

Many sectors use fibre-reinforced polymer composites as their primary material, which has led to substantial characterisation and understanding of their behaviour in harsh environments. However, it is important to know the different types of temperatures within or outside the Earth’s atmosphere, where the material response is drastically altered. Examples of applications are satellites, rockets, launch vehicle structures, aircraft structures at cruising altitudes, and glacial exploration structures (generally boats and ships) [171]. Sapi and Butler [171] studied the different levels of cryogenic temperatures and stated that a specific temperature does not define cryogenics; it is commonly referred to as −150 °C and occurs below the boiling points of nitrogen–oxygen, hydrogen, and helium. Table 6 shows the investigation of the cryogenic and low temperatures of composites, where the range of cryogenic temperature is −273 °C (0 K) to −150 °C (123 K), low temperature is −150 °C (123 K) to −50 °C (223 K), and room temperature is around 23 °C (RT).

In addition, the mechanical characteristics of composite materials have been studied extensively at various ambient temperatures. Some investigations were concerned with composite material impact behaviour [172,173]. Ma H et al. [174] investigated low-velocity impact tests with 8.44 J energy levels on glass-fibre/epoxy-polymer composites evaluated at various temperatures, such as ambient temperature (295 K), dry ice temperature (199 K), and liquid nitrogen temperature (100 K). Their study found that when the temperature drops, the material becomes more brittle, resulting in fewer areas of damage. In addition, they noticed that room-temperature samples suffered from severe fibre breaking and a larger overall damage depth compared to cryogenic temperature samples. Torabizadeh and Shokrieh [175] studied the effect of low temperatures (30 °C, −15 °C, and 23 °C) on the impact tests of glass-fibre-reinforced epoxy composites. They observed that the maximum absorbed energy decreases by about 25% when the temperature is lowered from room temperature. Besides, specimens that have been exposed to low temperatures for 10 days exhibit lower impact-energy absorption (about 10%) than specimens that have been exposed for one day at the considered temperatures. Salehi-Khojin et al. [176] examined the effect of temperature (−50 °C to −120 °C) on the impact properties of GFRP laminates. They noted that the laminates became rigid with high stiffness at low temperatures, resulting in only tiny deflections during impact testing. Even Icten et al. [177] also observed similar findings in GFRP laminates. They found that low temperatures at −60 °C and 20 °C had smaller damaged areas and a more significant perforation threshold subjected to a low-velocity impact test, whereas Ibekwe et al. [178] investigated the effects of low-velocity impacts and compressive after-impact tests at low temperatures between 20 and −20 °C on unidirectional and cross-ply glass–epoxy laminates. It has been found that temperature significantly affects the impact resistance of laminated composites. Additionally, they found that specimens with decreasing temperatures caused more damage to the composites. However, in carbon-fibre-reinforced composites (CFRP), Rio et al. [179] examined the low-velocity impact response of unidirectional, cross-ply, quasi-isotropic, and woven carbon–epoxy laminates at low temperatures. The results of the experiments revealed a 50% drop in threshold energy in quasi-isotropic laminates when the temperature was reduced from 20 °C to −150 °C. Furthermore, no traces of damage were seen on the laminates. In a similar approach, Kwang-Hee et al. [180] investigated impact damage in CFRPs down to −30 °C, and López-Puente et al. [181] extended this investigation to −150 °C. They both concentrated on high-velocity perforating impacts (from 100 to 500 m/s), far beyond the threshold impact energy. In addition, when perforation occurs, the impact is hugely confined to the contact area, resulting in a reduced delamination extension.

**Figure 14:**
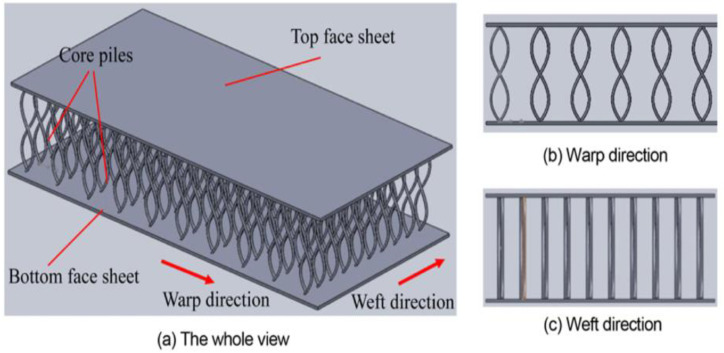
3D-integrated woven spacer composite structure diagram (**a**) the whole view, (**b**) warp direction (**c**) weft direction [183] (reprinted with permission from Springer Nature, Licence Number:5447071147988).

**Figure 15:**
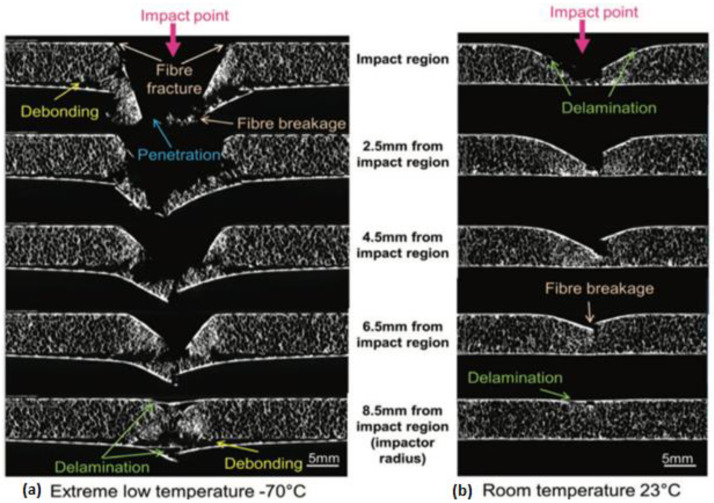
μCT scans of specimens impacted with 10 J impact energy at (**a**) extreme low temperature −70 °C and (**b**) room temperature 23 °C [182] (reprinted with permission from SAGE Publications, Licence Number: 5447071365227).

Some studies have demonstrated improved impact behaviour at low and cryogenic temperatures; however, these studies focused on three-dimensional integrated woven sandwich structures, tubes, and multiaxial warp-knit or stitched laminates with enhanced impact properties [182,183]. Li et al. [183] investigated the impact failure of a 3D-integrated woven composite at room or cryogenic temperatures. Their study focused mainly on the core heights of the laminates (refer to Figure 14). Their experiments showed that the impact energy of the composites increased with increasing core height, both at room temperature and in liquid nitrogen. In addition, the authors further noticed that when compared to room temperature, the liquid-nitrogen temperature significantly enhances the impact properties. However, Khan et al. [182] had a different opinion on extremely low and room temperatures. They stated that specimens impacted at a very low temperature (−70 °C) have less strength and are more prone to damage. Figure 15a shows that the impact region undergoes complete penetration, even at low temperatures of −70 °C. However, the impact region is not entirely penetrated at 23 °C (refer to Figure 15b). This is because the sandwich panel performs more brittlely at extremely low temperatures, such as −70 °C, than at 23 °C. Furthermore, another study also revealed precisely the same result in CFRP [184]. The authors Mohammed Elamin et al. [184] stated that the arctic low-temperature environment (−70 °C) significantly impacted composite strength and caused complex damage mechanisms. Due to the increased strength of the fibres at cryogenic temperatures, the load-bearing role of the brittle resin with low ductility is less affected in the out-of-plane direction. In addition, as compared to thermoset materials, thermoplastic resins can enhance absorbed energy and impact strength [185,186].

Moreover, there are no detailed studies on the influence of cryogenic temperatures in NFRCs. However, there are few studies reported on natural fibres. Sarasini et al. [187] examined the effect of temperature in the basalt- and glass-fibre-reinforced thermoplastic fibre–metal laminates subjected to low-velocity impact tests. Low (−30 °C) and room temperatures were performed in this study. It was found that the basalt-based laminates continued to exhibit higher peak forces and deformations than glass-based laminates, even if the lower temperature substantially reduced deformation abilities. Vinod and Sudev [188] examined the effect of cryogenic temperature on jute- and hemp-fibre-reinforced polymer composites. They found that the composite’s maximum impact strength at room temperature was 8.935 kJ/m^2^. In addition, they noticed that temperature drops cause a large number of tiny cracks to form within the composite material, causing the material to become brittle and resistant to unexpected loads, reducing its toughness and impact strength. A detailed summary of the effect of low and cryogenic temperatures on the impact behaviour of composites is illustrated in Table 7.

#### 4.2.2. Effects of High Temperatures

Many authors have studied the effect of temperature on the impact behaviour and damage tolerance of polymer-matrix-reinforced composites since matrix ductility and toughness are increased at high temperatures. Most studies depict glass-fibre- [195,196,197,198] or carbon-reinforced [199,200,201,202] composites in the literature, but more recently, hybrid reinforcements in hemp–basalt- [203] and Kevlar/glass-reinforced composites [176] have been investigated. Epoxy-based composites are mentioned in several references [195,196,197,198]; however, very few refer to thermoplastic laminates [180,199]. In an experiment comparing carbon/epoxy and carbon/PEEK laminates, Im et al. [180] tested the temperature-induced damage on orthotropic laminates. They found that impact-induced delamination decreases with increasing temperature. Results made from PEEK laminates had lower transverse crack frequencies than epoxy laminates. In addition, Biboka et al. [199] evaluated different matrix types and morphologies on the composite’s ability to absorb energy, resist penetration, and resist damage caused by different temperatures. With high energy, the indenter penetrates the specimen completely. An impact at low velocity induces damage, but the plate remains intact. However, the epoxy-based laminates were more prone to delamination at high-temperature tests when subjected to impacts at low speeds.

Furthermore, Biboka et al. [204] conducted another impact-damage study on residual CAI properties at extreme temperatures. They observed that testing temperature significantly affects CAI strength, whereas the impact temperature only had a marginal impact. In addition, it is known that the delamination growth during compression is constrained at high temperatures in the thermoset-hardened epoxy but not at ambient temperature in the thermoplastics PAS (polyarylsulfone). Sorrentino et al. [205] studied temperature influence on carbon fibre reinforced with thermoplastic polyethene-naphtholate (PEN) composites. The impact and flexural behaviour were evaluated at different temperatures, and it was found that the temperature rise enhances the impact properties of C/PEN/laminates. Besides, the presence of T_g_ (glass transition temperature) of the composites had a minimal influence on flexural rigidity and low-impact resilience.

Regarding NFRCs, only a few studies are related to the high temperatures subjected to impact tests. Rajaei et al. [206] tested glass and flax composite laminates with low speeds and found that glass–epoxy laminates at 300 °C maintained a lower peak load in the impact tests, whereas the flax–epoxy laminates had lower energy absorption and lower deflection because of the poor weakening of the fibres. Suresh Kumar et al. [203] noted that increases in temperatures could damage the impact properties on hemp/basalt fibres. Further observation found that hemp and hybrid/epoxy composites performed better at 50 °C than basalt/epoxy composites. Mueller [207] observed that all composites, irrespective of fibre type, showed similar performance with a maximum impact strength in the medium temperature range. Dhakal et al. [14] studied the effect of temperature and impacted velocity on jute-unsaturated polymer composites (UP). They found that jute/UP specimens exhibited the highest percentage of the original strength at 30 °C and 50 °C compared to the 75 °C specimens tested. In addition, a study by Shen and colleagues [162] noticed the same results and found that moderately high temperatures could reduce the impact damage of flax-fibre composites. David-West et al. [208] observed natural-fibre–polystyrene composites exhibit a certain degree of plasticity at higher temperatures. A sudden drop in load was observed in flax-fibre composites when temperature tests were carried out at 40 °C and 60 °C, respectively. Possibly this could be due to a loss in stiffness and energy accumulated in the composites, which later may be dissipated. A study by Singh et al. [209] compared the curing effect of high and low temperatures on NFRC samples. Using experimental results, the authors discovered that changes in curing temperature lower impact strength but increased tensile and flexural strength. The changes in those strengths reduce flexural and tensile strength, reaching a maximum of 100 °C. A detailed summary of the effect of high temperatures on the impact behaviour of composites is illustrated in Table 8.

## 5. Ways to Minimise the Moisture Ingress and Its Influence on the Impact Characteristics

Various ways to minimise moisture ingress and the influence of natural-fibre composites are discussed in the following sections.

### 5.1. Hybrid Technique

During the past few years, NFRCs have rapidly increased because of environmentally sustainable benefits over synthetic fibres. Besides, these benefits include biodegradability, recyclability, low energy consumption, and low weight [210,211,212,213]. However, these composites have several drawbacks, such as incompatibility with the reinforcements and high sensitivity to humidity and moisture [162]. In addition, the effect of water molecules on composites affects their mechanical, impact, and viscoelastic properties, leading to degradation [214,215]. Hence, it becomes necessary to modify the fibre structure so that moisture can be reduced in the fibre while retaining its high thermo-mechanical properties. Another possible strategy for improving moisture uptake and obtaining good mechanical and viscoelastic properties in NFRCs is hybridisation [216]. It combines two or more fibre types, natural–natural or natural–synthetic, simultaneously in a polymer matrix. Hybrid polymeric composites have superior properties to conventional composites [217,218]. In addition, the sensitivity associated with moisture uptake is also decreased. Hybridisation with two or more natural fibres is more environmentally friendly than synthetic elements [219].

**Figure 16:**
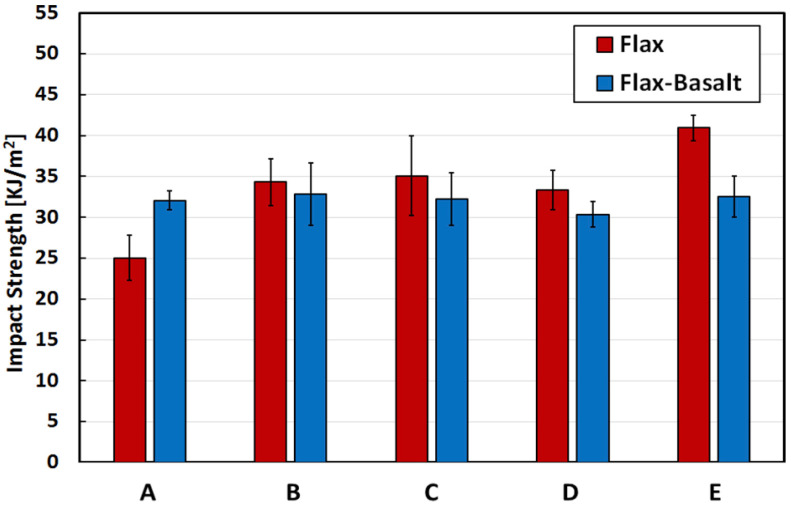
Impact strength for flax and flax–basalt composites [226] (reprinted with permission from Elsevier, Licence Number: 5447080120537).

Numerous studies have been conducted on composites in relation to their impact toughness, water absorption, and other mechanical characteristics. A natural fibre has many hydroxyl groups on its surface, making it highly sensitive to water molecules [220], whereas synthetic fibre such as carbon or glass has better hydrothermal ageing resistance. Hybridising natural fibres with synthetic fibres can enhance the durability of the composites. In addition, these fibres could be used as exterior protective materials for NFRCs. According to Al-Hajaj et al. [221], carbon fibre improves the hydrothermal ageing behaviour of FFRCs by lowering the flax-fibre content and providing a barrier to water molecules. Dhakal et al. [222] found that the amount of absorbed water is significantly reduced when hybridising with carbon fibres. In another study, Almansour et al. [223] stated that fibre hybridisation with basalt improved the endurance of NFC because the basalt offered better protection to the swelled flax fibres. Živković et al. examined the effect of moisture absorption on impact properties using basalt and flax fibre reinforced and hybridised with vinyl ester composites [224]. They found that FFRCs absorbed more water (5.92%) than basalt-fibre-reinforced composites (0.70%). As a result of increased ductility, flax fibre showed greater impact resistance after accelerated ageing. Alternatively, basalt protection produced the highest fibre/matrix adhesion and lowest moisture intake [225]. On the other hand, Fiore et al. [226] achieved comparable results. They noticed that impact findings revealed a significant difference in behaviour between flax and flax–basalt composites, as shown in Figure 16. Because of the existence of exterior basalt layers in the hybrid structures, the impact strength of unaged flax–basalt samples are 28% higher than flax samples. Flax–basalt composites do not show substantial variations in impact strength, but flax composites enhance their energy absorption capabilities as the ageing period increases. In another article, Fiore et al. [227] recently assessed the moisture absorption behaviour of hybrid flax–glass–epoxy-reinforced composites in salt-fog environments. They observed that the stacking sequence of the outer glass-fibre-reinforced laminae protects the inner hydrophilic laminae reinforced with flax fibres, extending the material’s service life.

### 5.2. Influence of Various Surface Treatments

NFRCs are influenced by many parameters, such as interfacial bonding, composition, and the matrix’s toughness [228]. A major issue that could occur because of the distinct chemical structures of the plants and polymer matrix is poor coupling between the two phases, resulting in insufficient stress transfer at the composite interfaces [229]. In fibre-reinforced composites, the interface plays a vital role in determining strength and toughness [93]. A disadvantage to using natural fibres in a polymer matrix is their high water-absorption rate, resulting in high swelling, degradation, and poor fire and chemical resistance [230]. However, these composites exhibit relatively low fibre–matrix adhesion, which, if enhanced, would eventually eliminate all listed constraints while improving mechanical properties [231].

According to the literature available, the surface treatment of natural fibres improves flexural strength and tensile strength but reduces impact strength. As per Bledzki et al. [232], adding maleic-anhydride-grafted polypropylene (MAPP) to the matrix phase reduces the impact toughness of the composite material because of increased brittleness in the matrix. In another study, Mehta et al. [233] stated that the chemical treatment of silane, acrylonitrile, and methyl ethyl ketone peroxide (MEKP) in hemp fibre enhanced the impact strength of fibre laminates compared to untreated fibre laminates. Among all treatments, acrylonitrile produced the best results in terms of increasing impact strength. They also observed that the value of impact strength for NFRCs was influenced not only by the sort of chemical treatment utilised to treat the fibres but also by the type of natural fibre. In addition, the authors conducted further study on sisal fibres treated with silane treatment. They noticed that sisal fibres treated with silane agents had a detrimental effect on impact strength, whereas hemp fibres treated with silane agents positively affected impact strength. In a similar study, Sree Kumar et al. [234] found alkali treatment had the most negative influence on the sisal/PE composite’s impact strength compared to silane treatment. However, in another contrast study, Thiruchitrambalam et al. [235] reported that in comparison to alkali treatment, sodium lauryl sulphate treatment (SLS) of banana/kenaf fibre improved the impact strength of a banana/kenaf hybrid composite. Dayo et al. [236] observed that after chemical treatments, hemp-fibre-reinforced polybenzoxazine composites had better mechanical properties than untreated and alkali-treated hemp-fibre/polybenzoxazine composites. A study by Sreekala et al. [237] explored the effect of chemical treatments and impact resilience on palm-fibre/phenolic formaldehyde composites. They found that latex treatment achieved superior resilience on impact tests, whereas peroxide treatment yielded the slightest improvement. Shanmugam et al. [238] found that with increasing percentages of jute fibre in the composite, impact strength values decreased owing to improved stress transfer from fibre to the matrix. A further observation was undertaken by Venkateshwaran et al. [239] on banana-fibre-reinforced epoxy composites treated with alkali. They highlighted that 1 wt.% NaOH in the alkali treatment of banana fibres gave the maximum value of impact strength of banana/epoxy composites in contrast to those treated with 0, 2, 5, 10, 15, and 20 wt.% NaOH. Karthikeyan et al. [240] claimed that alkalisation increases the impact strength of coir-fibre composites. Various concentrations of alkalising agents were used for 10 days, i.e., 2% to 10%. The alkalisation/mercerisation process roughens the surface of the fibre. As more surface area becomes available, better bonding between the fibres and matrix occurs, leading to greater mechanical strength.

Based on the above literature studies, it is evident that most chemical treatments have detrimental effects on the impact strength of NFRCs. As opposed to improving tensile and flexural strength, enhancing IFSS in NFCs is not always beneficial to impact strength. It has been found that the most prevalent chemical treatment, mercerisation, has a negative impact on the impact strength of NFCs. In contrast, only a few chemical treatments, such as silanisation, latex treatment, and MEKP treatment, are beneficial in improving the impact strength of NFRCs. Table 9 depicts the different chemical treatments and their effects on various natural fibres.

## 6. Applications of NFRCs

Compared to synthetic-fibre-reinforced composites, NFRCs are less environmentally harmful. Natural plant fibres are promising for industrial applications because they are biodegradable, lightweight, cost-effective, and environmentally friendly [255]. However, NFRCs, in particular, are being encouraged by researchers in this context [256]. Natural plant fibres such as hemp, flax, and kenaf are used in various applications such as aerospace, automotive, marine, construction, and packaging [257]. The following section presents and discusses applications for natural fibres across various industries.

### 6.1. Marine Industries

**Figure 17:**
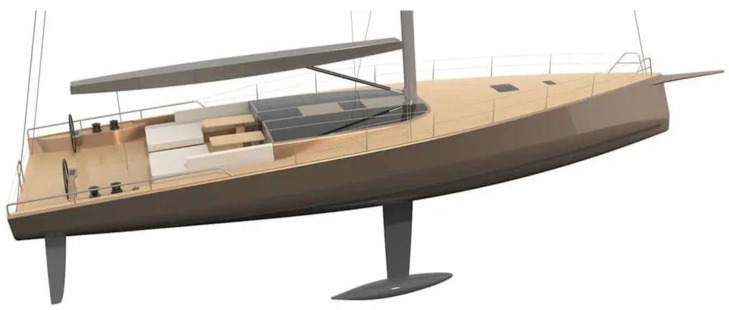
Baltic yachts made from flax-fibre-reinforced composites (source: https://www.metstrade.com/news/construction-and-material/flax-basalt-fibres-future-marine-composites/, 5 July 2020) [261].

Synthetic fibres like carbon-fibre-reinforced composites are the most common material used in the construction of small boats, which shows good long-term performance in these conditions [258]. However, considering natural fibres’ sensitivity to water, the matrix polymer must provide long-term protection if these composites succeed as glass-fibre replacements in the marine environment [219]. Despite their environmental stability, natural-fibre composites have recently been introduced as an eco-friendly alternative for making boats and surfboards. As the composite hull structure is continuously exposed to seawater, the water-resistant properties of these materials are essential [259]. In today’s scenario, sails, ropes, and boats have been made from natural plant fibres for marine applications [260]. More recently, the Amer yacht company [261] built rigid inflatable boats, and high-end-performance Baltic yachts, shown in Figure 17, made from flax-fibre-reinforced composites as an alternative to carbon fibre. In addition to other sustainability objectives, Baltic yachts have determined that flax fibre is an excellent choice for further development since it is a naturally grown and readily available plant-based material.

### 6.2. Aviation Industries

Currently, aircraft industries are aiming to manufacture interior aircraft components like air ducts, ceiling panels, seat end caps, and other non-load-bearing parts using natural plant fibres like hemp, flax, and kenaf [262]. However, in one of the articles, authors Alonso-Martin et al. [263] investigated the natural-fibre-based thermoset and thermoplastic skins for manufacturing aircrafts’ interior panels. They stated that a typical commercial aircraft saves 0.02–0.04 kg of fuel per hour for each kilogram of weight reduction using thermoset and thermoplastic skins based on natural fibres. Additionally, they further observed that thermoplastic resin panels reduce CO_2_ emissions because of their weight reduction. In 2021, Boeing Research and Technology Europe (Madrid, Spain) collaborated on a European project called Cayley, which focussed on sustainability and “green” interiors [264]. The project aimed to industrialise interior panels using renewable polymers from recyclable thermoplastic sheets and natural fibres, namely flax, where they reported that compared to carbon-fibre/epoxy prepreg tapes, FlaxPreg linen/epoxy prepreg tapes are 35% lighter.

Aircraft exterior parts, like the cockpit, wings, flaps, and rudder, are subjected to low-velocity impacts caused by bird strikes, hailstones, runway debris, and other factors [249]. However, it is imperative to understand how natural plant-fibre composites respond to impact damage, particularly at low-velocity impact events.

### 6.3. Automotive Industries

Natural-fibre composites are utilised in the automobile sector for lightweight construction, significantly decreasing greenhouse gas (GHG) and CO_2_ emissions [265]. According to research studies, NFRCs can lead to a 20% cost reduction and 30% weight reduction in vehicle components [266]. For example, a typical car produced in Germany contains 3.6 kg of natural-fibre parts, which is the most significant consumer of natural-fibre parts among all the European automotive industries [267]. The majority of natural-fibre composite parts are used for interior applications, such as dashboards, seat backs, and door panels, in contrast to exterior applications [268]. In 2019, Porsche planned to build a racing car with bodywork made from composites reinforced with natural plant-fibre-reinforced composites such as flax and hemp [269]. In recent years, new studies have been conducted on NFRCs, in which automobile hoods are made of flax and vinyl ester composites [270]. A brake pad comprises palm kernel shell fibre and phenolic resin [271], and the door panels are made of bamboo/polyurethane composites [272]. Therefore, as a marketing advantage, most automotive manufacturers currently use NFRCs to reduce vehicle weight, costs, and their life-cycle impact on the environment.

## 7. Future Prospects

The use of natural fibres in polymer composites is becoming a viable and sustainable alternative to glass fibres because of their low cost, low weight, and eco-friendly properties. NFRCs are employed in various industries, including automotive, electrical, construction, and home appliances. As a generalisation, all plant-fibre composites absorb moisture in humid environments, which eventually causes the fibre/matrix interface region to degrade, resulting in distortion in dimensional properties and poor stress transfer. Besides, several variables affect moisture intake, such as fibre contents, humidity, matrix, temperature, and moisture distribution within the composite. As part of assessing the physical properties of composites under different environmental conditions, environmental characterisation of NFRCs has emerged as a significant dimension, which requires considerable effort. Therefore, based on the extensive literature review, this review paper has discussed a broad range of research on the environmental influences of NFRCS by focusing more on the moisture uptake of plant fibres and their effects on the polymer matrices and their enhancement in the composite laminates subjected to the impact tests of low, high, and cryogenic temperatures. Different impact characteristics were discussed and highlighted. However, a few gaps were further highlighted and need to be addressed in the NFRCs that are not yet entirely focussed on the research studies. They are:

1. It is essential to know the impact behaviour of natural fibre embedded with polymer matrixes subjected to different humid conditions. However, in the current literature studies, the work focused on the effects of varied environmental conditions on the tensile, flexural, and other mechanical properties of natural-fibre composites. Therefore, further research is still needed on the impact behaviour and shear responses of natural composites with varying RH values.
2. In addition, there are no sufficient studies on the effects of high, low, and cryogenic temperatures on the impact damage behaviour of NFRCs. However, it is essential to know how natural-fibre laminates behave in harsh environments, which could be helpful for many engineering applications. Particularly in marine sectors, natural-fibre materials are being tested for use in designing and building new boats for glacial exploration structures.
3. Based on the above literature studies, it is evident that most chemical treatments have detrimental effects on the impact strength of NFRCs. As opposed to improving tensile and flexural strength, enhancing IFSS in NFCs is not always beneficial to impact strength. It has been found that the most prevalent chemical treatment, mercerisation, has a negative impact on the impact strength of NFCs, whereas only a few chemical treatments, such as silanisation, latex treatment, and MEKP treatment, are beneficial in improving the impact strength of NFRCs. Therefore, further research is still required on enhancing the chemical modification of natural fibres, particularly for impact properties.

## 8. Concluding Remarks

In this review paper, the critical aspects of the effect of environmental conditions on the impact performance of natural-fibre-reinforced composites are discussed. In addition, this paper critically assessed the damage mechanisms of NFRCs and their hybrids by focusing more on moisture ingress and relative humidity in the impact damage behaviour of NFRCs. The research so far demonstrates the utilisation of natural fibres as a suitable replacement for synthetic-fibre-reinforced composites from a sustainable and ecological perspective. However, fundamental and technological problems must be addressed to use these fibres fully. For NFRCs to be used in structural and semi-structural applications, the fibre-hybridising approach has been considered one of the optimising techniques. This approach not only provides property enhancement opportunities but also provides a cost-effective way to minimise the drawbacks of NFRCs. Several critical factors influencing long-term durability and using natural fibre-reinforced hybrid composites in harsh environments are well-explained. Additionally, this review identifies and highlights the following crucial points.

- Moisture ingress significantly reduces the load-bearing capacity of NFRCs when exposed to harsh environments, particularly for sub-zero and high temperatures. In addition, the effect of moisture on the impact performance of natural-fibre composites is critical in engineering applications such as marine, automotive, and aerospace because it can modify the behaviour of the structure under varied loading conditions. As far as protection and withstand ability are concerned, fibre hybridisation significantly improves the moisture ingress and impact behaviour of natural fibre-reinforced composites.
- Bio-based plant composites will play a significant role in the future, where environmental credibility is of prime importance.

There is a growing market for biocomposites, which are described as a novel application. However, it is worth noting that natural plant fibres were developed for aircraft, automobiles, and marine applications. It would be easy to conclude that nothing is new, but that would be too simplistic. Undoubtedly, the composites industry has developed significantly over the past few decades, and this is evident in the gradual increase in composite materials used in structural applications. As biocomposites become more industrialised, the novelty comes from their increased industrialisation rather than their raw materials, which leads to increased competition. However, given that they use renewable resources, have minimal adverse effects on the environment, and offer end-of-life solutions that go far beyond the reasons that led to their initial development as structural materials 80 years ago, it is still possible to classify these biocomposites as materials of the future.

## Figures and Tables

**Table 1:** Mechanical and physical properties of commonly used natural fibres.

Fibre	Density (g/cm^3^)	Elongation (%)	Tensile Strength (MPa)	Elastic Modulus (GPa)	Length mm	Diameter μm	L/D	References
Cotton	1.5–1.6	7.0–8.0	400	5.5–12.6	20–70	20–30	1250	[34,35,36,37,38]
Jute	1.3	1.5–1.8	393–773	26.5	2–3	16	160	[34,35,36,37,38]
Flax	1.5	2.7–3.2	500–1500	27.6	2–40	20–23	100–2000	[34,35,36,37,38]
Hemp	1.47	2–4	690	70	5–60	20–40	100–2000	[34,35,36,37,38]
Kenaf	1.45	1.6	930	53	---	---	---	[34,35,36,37,38]
Ramie	N/A	3.6–3.8	400–938	61.4–128	40–150	30	40–150	[34,35,36,37,38]
Sisal	1.5	2.0–2.5	511–635	9.4–22	2–7	20	140	[34,35,36,37,38]
Coir	1.2	30	593	4.0–6.0	---	---	---	[34,35,36,37,38]

**Table 2:** Chemical compositions of different bast fibres [42,43,44,45].

Fibres	Cellulose	Lignin	Hemicellulose	Pectin	Ash	Moisture Content	Wax
	%	%	%	%	%	%	%
Flax	71	2.2	18.6–20.6	2.3	-	8–12	1.5–3.3
Kenaf	31–72	15–19	21.5–23	-	2–5	-	-
Jute	45–71.5	12–26	13.6–21	0.2	0.5–2	12.5–13.7	0.5
Hemp	57–77	3.7–13	14–22.4	0.9	0.8	6.2–12	0.8
Ramie	68.6–91	0.6–0.7	5–16.7	1.9	-	7.5–17	0.3
Abaca	56–63	7–9	15–17	-	3	5–10	-
Sisal	47–78	7–11	10–24	10	0.6–1	10–22	-
Henequen	77.6	13.1	4–8	-	-	-	-

**Table 3:** Cross-sectional area variations of different types of natural fibres [47].

**Fibre**	**Average CSA** **(mm^2^)**	**95% Confidence Limit of the Average CSA**	
		Intra-fibre	Inter-fibre
Sisal	0.326	5.0%	24.3%
Coir	0.028	11.3%	24.0%
Abaca	0.021	6.5%	42.5%
Flax	0.012	7.5%	24.1%
Kenaf	0.006	12.9%	15.6%
Hemp	0.005	10.8%	27.6%
Jute	0.003	11.0%	18.3%

**Table 4:** Water ingress mechanism of pure and modified sisal composites [120].

Types Of Diffusion		Diffusion Exponent (*n*)	Time Dependence	Mechanism
Case I	First phase less Fickian Diffusion	*n* < 0.5 *n* = 0.5	$t^{-0.5}$ $t^{0.5}$	Water molecule diffusion occurs at a considerably slower rate than polymer segment mobility.
Case II	Case II Diffusion	*n* = 1.0	(Time-independent)	The diffusion process is far more active than the relaxing process.
	Super Case II Diffusion	*n* > 1.0	$t^{n-1}$	
Case III	Non-Fickian/ Anomalous Diffusion	0.5 < *n* < 1.0	$t^{n-1}$	Water-molecule mobility is equivalent to polymer-segment mobility, an intermediary performance between Case I and Case II diffusion.

**Table 5:** Different stages of thermal degradation of natural fibres.

First Stage	Second Stage	Third Stage	References
50–100 °C	200–300 °C	330–500 °C	
Moisture evaporation and fibre degradation happens due to the release of water absorbed by the fibres	Thermal decomposition happens for hemicellulose, lignin, pectin, and glycosidic linkages	Weight loss happens due to lignin and cellulose	[7,165,166]

**Table 6:** Different levels of cryogenic temperatures used in the polymer–matrix composites adapted from reference [171].

Type	Kelvin (K)	Celsius (°C)	Category of Temperatures
Normal room temperature	296	23	Room Temperature
The temperature of arctic conditions	223	−50	Low Temperature
Temperature for aircraft components	216	−57	Low Temperature
Carbon dioxide (dry ice)	195	−78	Low Temperature
Earth’s lowest temperature	184	−89	Low Temperature
Liquid nitrogen (LN_2_)	77	−196	Cryogenic Temperature
Liquid oxygen (LO_X_)	90	−183	Cryogenic Temperature
Liquid hydrogen (LH_2_)	20	−253	Cryogenic Temperature
Liquid helium (LHe)	4.2	−269	Cryogenic Temperature

**Table 7:** A detailed summary of the effect of low and cryogenic temperatures on the impact behaviour of fibre-reinforced composites.

Fibre	Structure	Matrix	Temperature [K]	Properties Compared to Room Temperature	References
Carbon	UD	Epoxy (R608)	77	Impact energy (I) increases	[189]
Carbon	QI and cross-ply laminates from UD	Epoxy (3501-6)	123	Absorbed energy (Eabs) increases	[179]
Carbon	Woven	Epoxy (8552)	123	Absorbed energy (Eabs) decreases, and low-velocity impact (I) energy increases	[179]
Carbon	UD	Vinyl Ester	173	Absorbed energy (Eabs) increases to 130 %	[190]
Carbon	UD	Vinyl Ester	223	Absorbed energy (Eabs) decreases, and low-velocity impact (I) energy increases	[191,192]
Glass	Woven	Vinyl Ester	223	Absorbed energy (Eabs) decreases, and low-velocity impact (I) energy increases to 3 %	[176]
Glass (E-glass)	Woven	Epoxy	223	Absorbed energy (Eabs) decreases, and low-velocity impact (I) energy increases	[193]
Glass (E-glass)	Woven	Epoxy	213	Absorbed energy (Eabs) decreases, and low-velocity impact (I) energy increases	[177]
Basalt	Chopped fibre	PP (HP 500M) + nano clay	77	Absorbed energy (Eabs) increases, and low-velocity impact (I) energy decreases to 8%	[194]

**Table 8:** Summary of the effect of high temperatures on impact behaviour of fibre-reinforced composites.

Fibre	Structure	Matrix	Temperature [°C]	Properties Compared to Room Temperature	References
Carbon T300-3000	Orthotropic	Epoxy	120	Delamination area decreases with impact energy	[180]
Carbon	Orthotropic	PEEK	120	Delamination area increases, but matrix cracking decreases	[180]
Carbon	Quasi Isotropic	Epoxy	150	Very few delaminations are observed	[204]
Carbon	Woven	Polyethene-naphtholate	100	Low-impact resistance + enhanced toughness	[205]
Flax	Woven	Epoxy	300	Poor impact resistance due to fibre weakening	[206]
Glass	Woven	Epoxy	300	Increased absorption + maximum deflection	[206]
Jute	Woven	Unsaturated polyester	75	Low impact damage was observed at 30 °C and 50 °C, compared with 75 °C	[14]
Flax	Stacked sequence	Epoxy	100	Low impact damage was observed at 100 °C	[162]
Flax	Stacked sequence	Styrene polyester	100	Lower impact strength but increased tensile strength and flexural strength.	[209]

**Table 9:** Chemical treatments of natural fibres and their effects.

Chemical Treatment	Effects	References
Alkaline	It enhances the bonding of the rough surface of the fibre and improves the mechanical properties	[241,242]
Silane	It increases the physiochemical property between fibre and matrix	[243]
Acetylation	It enhances the dimensional stability and reduces the hydrophilic nature of the fibre	[244]
Bleaching	It enhances the mechanical properties and thermal stability of the fibre	[245,246]
Benzoylation	It enhances mechanical strength and thermal stability and improves the hydrophobicity	[247,248]
Acrylation and acrylonitrile grafting	It improves the stress transferability and enhances the adhesion between fibre and matrix	[49,249]
Maleated coupling agents	It improves the fibre wettability by providing efficient fibre–matrix interaction	[250,251]
Permanganate	It improves the interfacial bonding between fibre and the matrix	[252]
Peroxide	It enhances the mechanical strength of the composites and improves the interfacial bonding between fibre and matrix	[253]
Graft copolymerisation	It increases the thermal properties and mechanical strength	[248]
Polymer coating	It increases the bonding between the fibre and the matrix	[254]

## Data Availability

All data, models, and code generated or used during the study appear in the submitted article.
